# Comparison of Subgenomic and Total RNA in SARS-CoV-2-Challenged Rhesus Macaques

**DOI:** 10.1128/JVI.02370-20

**Published:** 2021-03-25

**Authors:** Gabriel Dagotto, Noe B. Mercado, David R. Martinez, Yixuan J. Hou, Joseph P. Nkolola, Robert H. Carnahan, James E. Crowe, Ralph S. Baric, Dan H. Barouch

**Affiliations:** a Center for Virology and Vaccine Research, Beth Israel Deaconess Medical Center, Boston, Massachusetts, USA; b Harvard Medical School, Boston, Massachusetts, USA; c Department of Epidemiology, The University of North Carolina at Chapel Hill, Chapel Hill, North Carolina, USA; d Vanderbilt Vaccine Center, Vanderbilt University Medical Center, Nashville, Tennessee, USA; e Department of Pediatrics, Vanderbilt University Medical Center, Nashville, Tennessee, USA; f Department of Pathology, Microbiology, and Immunology, Vanderbilt University Medical Center, Nashville, Tennessee, USA; g Ragon Institute of MGH, MIT, and Harvard, Cambridge, Massachusetts, USA; h Massachusetts Consortium on Pathogen Readiness, Boston, Massachusetts, USA; University of Texas Southwestern Medical Center

**Keywords:** SARS-CoV-2, genomic RNA, subgenomic RNA, viral load, non-human primates

## Abstract

Respiratory virus challenge studies involve administration of the challenge virus and sampling to assess for protection in the same anatomical locations. It can therefore be difficult to differentiate actively replicating virus from input challenge virus. For SARS-CoV-2, specific monitoring of actively replicating virus is critical for investigating the protective and therapeutic efficacy of vaccines, monoclonal antibodies, and antiviral drugs. We adapted a SARS-CoV-2 subgenomic RNA (sgRNA) RT-PCR assay to differentiate productive infection from inactivated or neutralized virus. Subgenomic RNAs are generated after cell entry and are poorly incorporated into mature virions, and thus may provide a marker for actively replicating virus. We show envelope (E) sgRNA was degraded by RNase in infected cell lysates, while genomic RNA (gRNA) was protected, presumably due to packaging into virions. To investigate the capacity of the sgRNA assay to distinguish input challenge virus from actively replicating virus *in vivo*, we compared the E sgRNA assay to a standard nucleoprotein (N) or E total (both gRNA and sgRNA) RNA in convalescent rhesus macaques and in antibody-treated rhesus macaques after experimental SARS-CoV-2 challenge. In both studies, the E sgRNA assay was negative, suggesting protective efficacy, whereas the N and E total RNA assays remained positive. These data suggest the potential utility of sgRNA to monitor actively replicating virus in prophylactic and therapeutic SARS-CoV-2 studies.

**IMPORTANCE** Developing therapeutic and prophylactic countermeasures for the SARS-CoV-2 virus is a public health priority. During challenge studies, respiratory viruses are delivered and sampled from the same anatomical location. It is therefore important to distinguish actively replicating virus from input challenge virus. The most common assay for detecting SARS-CoV-2 virus, reverse transcription PCR (RT-PCR) targeting nucleocapsid total RNA, cannot distinguish neutralized input virus from replicating virus. In this study, we assess SARS-CoV-2 subgenomic RNA as a potential measure of replicating virus in rhesus macaques.

## Abstract (precis)

Developing therapeutic and prophylactic countermeasures for the SARS-CoV-2 virus is a public health priority. During challenge studies, respiratory viruses are delivered and sampled from the same anatomical location.

## INTRODUCTION

**FIG 1:**
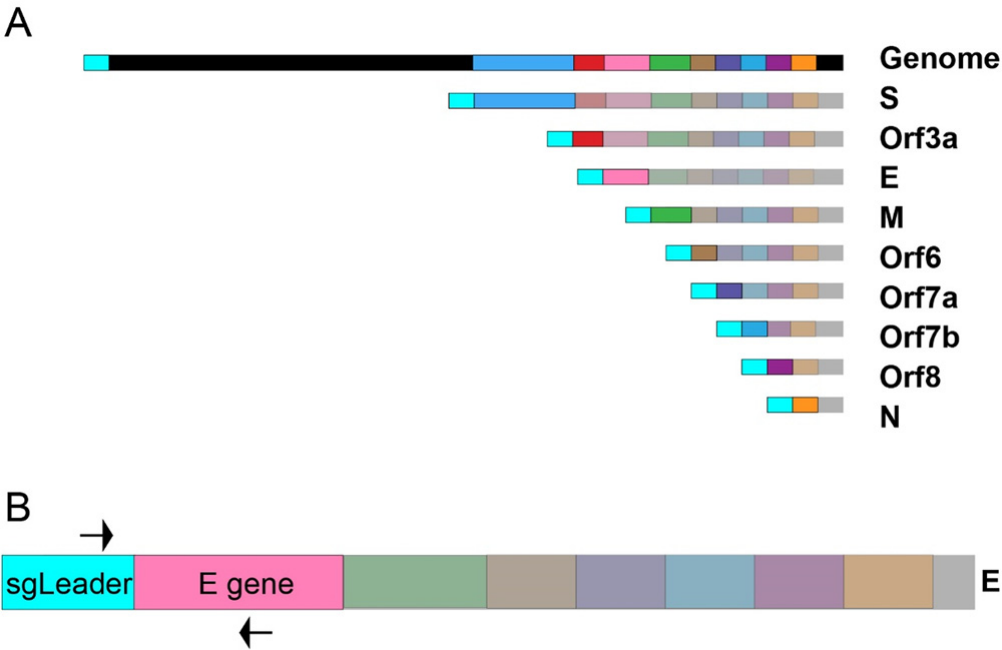
Graphical representation of sgRNAs and the E sgRNA assay. (A) Graphical representation of SARS-CoV-2 virus and sgRNA. Upon cellular entry SARS-CoV-2 generates sgRNAs for structural genes and accessory proteins before they are produced. The subgenomic leader sequence is colored cyan to highlight its position in the genomic and subgenomic RNAs. (B) Graphical representation of the primer binding sites for the E sgRNA assay on subgenomic E RNA. The forward primer binds to the subgenomic leader sequence present on all subgenomic RNAs as well as the genomic RNA. The reverse primer binds to the E gene (pink).

Members of the *Coronaviridae* family cause a wide range of respiratory and enteric diseases, ranging from mild illness to life-threatening infection. This family contains the largest known RNA viral genomes, ranging from 26 to 32 kb long (1). Coronaviruses utilize a positive-sense, single-stranded RNA genome that encodes several nonstructural and structural proteins. Two large polyproteins termed ORF1a and ORF1b encode nonstructural proteins that form the replication-transcription complex (2). The 3′ third of the genome consists of the main structural proteins: envelope (E), membrane (M), nucleocapsid (N), and spike (S), as well as other accessory proteins (2). The nonstructural genes are translated upon cytoplasmic entry, but the structural proteins must first be transcribed into subgenomic RNAs (sgRNAs) prior to translation (3). These sgRNA sequences consist of the leader sequence, the transcriptional regulatory sequence (TRS), and the target subgenomic gene followed by the rest of the genome 3′ of the gene. Subgenomic transcripts are thought to be generated through a discontinuous transcription model (4, 5). Negative-sense sgRNA transcription proceeds 3′ to 5′ from the 3′ end of the genome. Transcription continues until the first TRS preceding each subgenomic gene is reached. At this point, a fixed proportion of replication transcription complexes (RTCs) will continue transcription while the rest will stop transcription and transfer to the 5′ end of the genome (this is repeated for every subgenomic TRS) to add the leader sequence located at the 5′ end of the genome to the subgenomic transcript. This transfer is guided by the complementarity of the TRS sequence on the 3′ end of the nascent transcript and the TRS site proceeding the leader sequence in the 5′ end of the genome. Positive-sense sgRNA transcripts are then directly transcribed from the negative-sense sgRNA transcript (4, 5). In general, the viral sgRNAs are expressed in abundance relative to their proximity to the 3′ end of the genome, such that E sgRNA is much less abundant that N sgRNAs in infected cells (2). Such a method of transcription results in the generation of a set of nested sequences (Fig. 1A) (1, 4).

In December 2019, a novel SARS-like coronavirus emerged (6–8) and SARS-CoV-2 quickly spread throughout the world, resulting in a global pandemic (9). Phylogenetic analysis determined SARS-CoV-2 to be a member of the *Betacoronavirus* genus containing SARS-CoV (10). Determining the efficacy of candidate vaccines and therapeutics is therefore critical. Quantitating virus genome copy numbers from infected samples has been a reliable way to measure viral load (11, 12). Animal or patient samples are typically reverse transcribed (in the case of RNA viruses) and probed with virus-specific primer/probe sets by quantitative PCR (qPCR) to determine viral genome copy numbers (13). This method has also been used in previous outbreak virus vaccine studies, such as for Zika virus (14). Viral load assays were rapidly developed for SARS-CoV-2 infection monitoring, where the most prominent assay detects total RNA containing the N gene (15).

As a respiratory virus, SARS-CoV-2 poses a unique set of challenges concerning vaccine studies. Preclinical studies typically include viral challenges in the respiratory tract, typically by the intranasal and intratracheal routes. Monitoring of infection following challenge uses samples from the same anatomic locations, typically bronchoalveolar lavage fluid, nasal swabs, and respiratory tract tissues (16). An assay targeting total RNA or genomic RNA (gRNA) would presumably detect both input challenge virus, as well as newly replicating virus, and would not be able to differentiate between them. Thus, monitoring total RNA or gRNA following challenge may not be an optimal measure of protective efficacy.

A potential solution to this problem would be to assess sgRNA instead of gRNA. Subgenomic RNAs are only generated during productive infection and thus should present a more accurate measure of replicating virus. A sgRNA assay was originally described by Wölfel et al. (2020) (17), and we developed this assay for use in SARS-CoV-2-challenge studies in rhesus macaques (16). This assay has also recently been used by other groups conducting vaccine/challenge studies in rhesus macaques (18–20), making it critical to understand how subgenomic RNA differs from total RNA in the model. In this paper, we demonstrate the importance of targeting subgenomic RNA to differentiate productive infection from neutralized input virus in treated rhesus macaques.

## RESULTS

### E sgRNA specificity.

**FIG 2:**
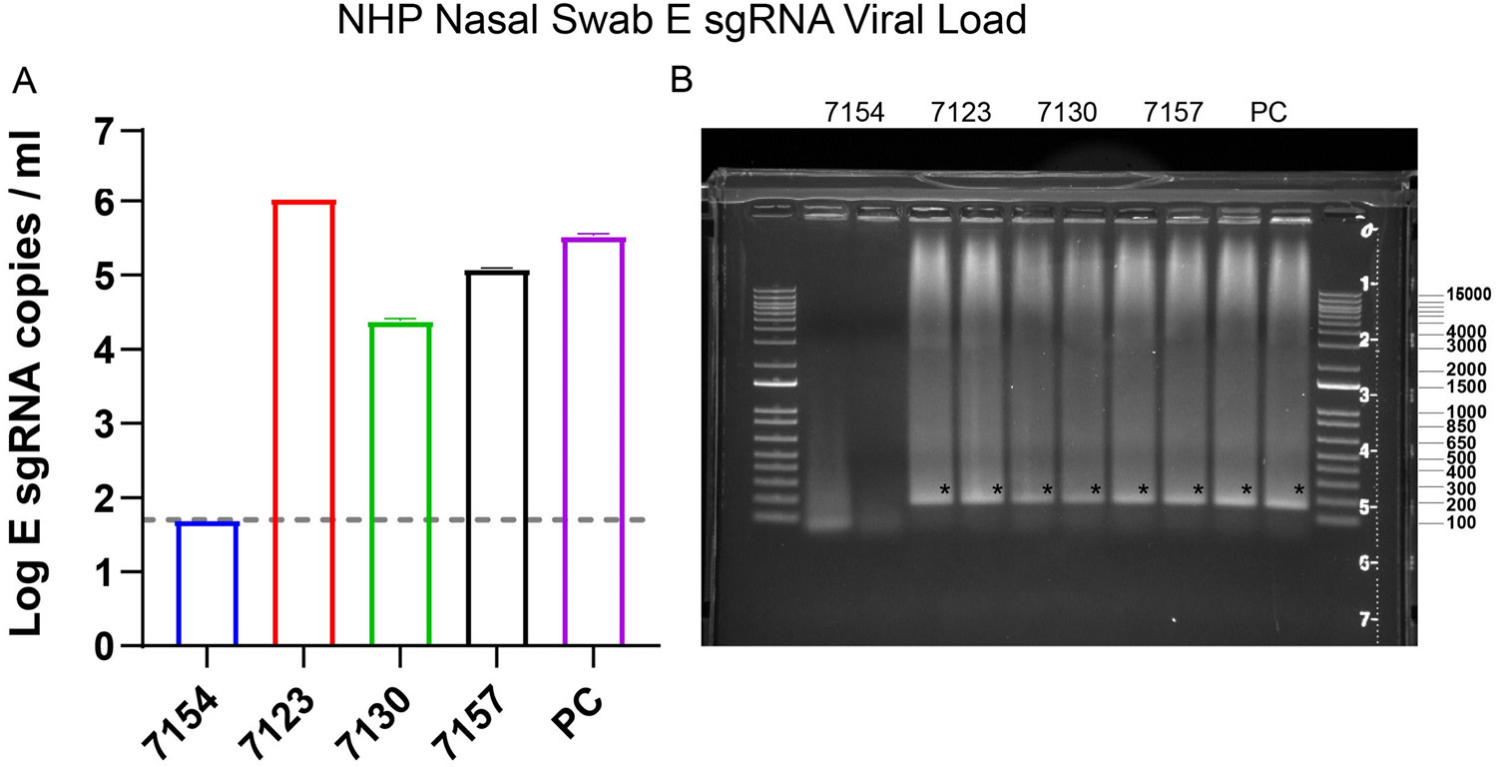
SARS-CoV-2-infected NHPs were sampled through nasal swabs on day 4 postinfection. (A) RNA was extracted from the nasal swabs and an E sgRNA RT-PCR assay was performed. (B) The assay RT-PCR results were then run in duplicate on a 0.8% agarose gel to confirm a single amplicon. Error bars define the standard deviation of the mean of two technical replicates for each macaque. PC indicates positive control. Asterisk indicates expected band.

After SARS-CoV-2 enters cells, a nested series of sgRNAs are generated (1, 4). The sgRNA RT-PCR assay was designed to target E sgRNA. We utilized a forward primer targeting the subgenomic leader sequence and a reverse primer and probe specific to the E gene (17). These primers span the junction between the subgenomic leader sequence and the E gene, thus providing high selectivity for E sgRNA (Fig. 1B). To demonstrate the specificity of this assay, qPCR products from SARS-CoV-2-infected macaques were run on an agarose gel (Fig. 2). The resulting gel had a single band for all positive samples at the expected size for the target amplicon (179 bp). Positive macaque qPCR amplicons were the same size as the E sgRNA positive control, further confirming assay specificity. The bands were sequenced and found to match the expected target amplicon.

**FIG 3:**
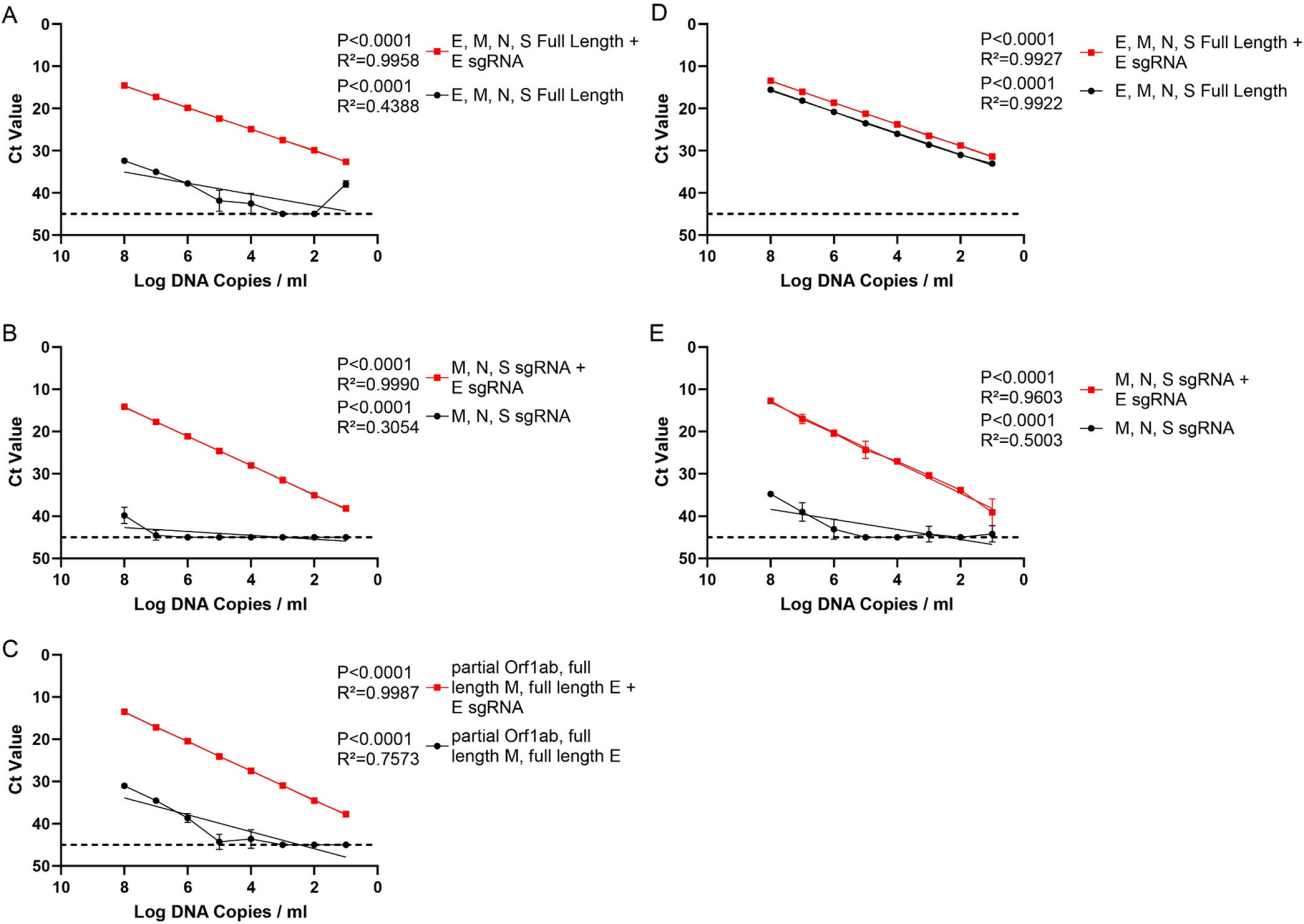
Assay specificity with linear DNA mixtures. RT-PCR was performed on DNA fragment mixtures with and without the addition of E sgRNA linear DNA fragments. These mixtures were serially diluted 10-fold from 10^8^ to 10 copies per ml. (A) Mixture of E, M, N, and S full-length DNA fragments. (B) Mixture of M, N, and S subgenomic partial DNA fragments. (C) Mixture of E and M full-length DNA fragments and the 5′ end of Orf1a containing the subgenomic leader sequence. In all mixtures, linearity was only present after the addition of E sgRNA. RT-PCR targeting E gRNA was performed on DNA fragment mixtures with and without the addition of an E sgRNA DNA fragment. (D) Mixture of E, M, N, and S full-length DNA fragments. (E) Mixture of M, N, and S subgenomic DNA fragments. Error bars denote the 95% confidence intervals of the mean of eight technical replicates. Lines represent simple linear regressions.

In order to confirm the E sgRNA primer/probe set targets only E sgRNA, we designed DNA fragments of multiple SARS-CoV-2 structural and nonstructural genes. Mixtures of DNA fragments with and without DNA corresponding to E sgRNA were evaluated by qPCR using the E sgRNA primer/probe set. Three different mixtures were generated testing E sgRNA specificity against the full-length (Fig. 3A) and subgenomic structural genes (Fig. 3B), as well as a gRNA fragment which contains a 5′ subgenomic leader sequence (Fig. 3C). Specific amplification over a 6-log dilution range was only observed in the presence of DNA corresponding to E sgRNA. As a control, qPCR assays for E gRNA amplified both mixtures (Fig. 3D and E).

### Lack of RNA amplification in virions by the sgRNA assay.

The E sgRNA assay should only amplify transcripts in the setting of active virus replication that produces sgRNA and should not amplify genomic RNA (gRNA). Laboratory virus stocks are typically cell lysates, which contain predominantly gRNA but also sgRNA from virus replication in cells. We therefore treated cell lysates with RNase A to degrade unpackaged RNA, but capsid-packaged gRNA should be protected.

**FIG 4:**
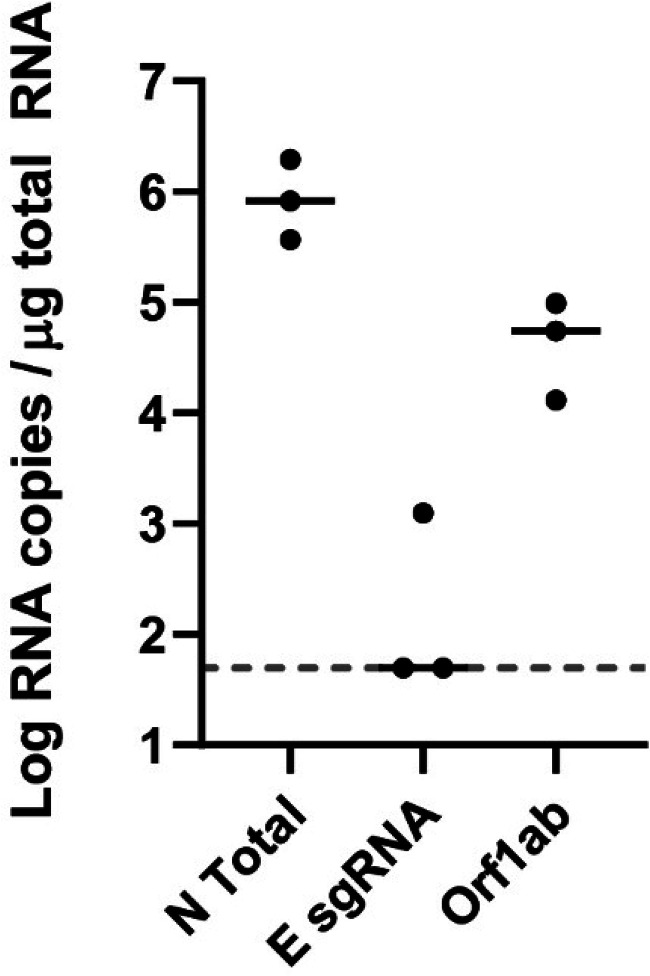
Infectious cell lysate treated with RNase A. Infectious cell lysate was treated with RNase A for 1 h and then RNA was extracted and RT-PCR for the N gene (N total), subgenomic E (E sgRNA), and genomic RNA (Orf1ab) was performed. Black bars represent median responses.

We extracted RNA from the RNase A-treated infection lysate and performed RT-PCR for the N total RNA, E sgRNA, and the Orf1ab gene that includes only gRNA, since Orf1ab does not generate subgenomic transcripts (21). After RNase A treatment, the median E sgRNA signal was at the limit of detection. The median Orf1ab and N total viral loads were >10^4^ and >10^5^ RNA copies per μg RNA, respectively (Fig. 4). The difference in N total and Orf1ab could be due to insufficient RNase A levels or trace amounts of N sgRNA packaged into virions (22). These data demonstrate that the E sgRNA assay does not detect genomic SARS-CoV-2 RNA in RNase-treated virions.

### Measuring sgRNA and gRNA during infection *in vitro*.

**FIG 5:**
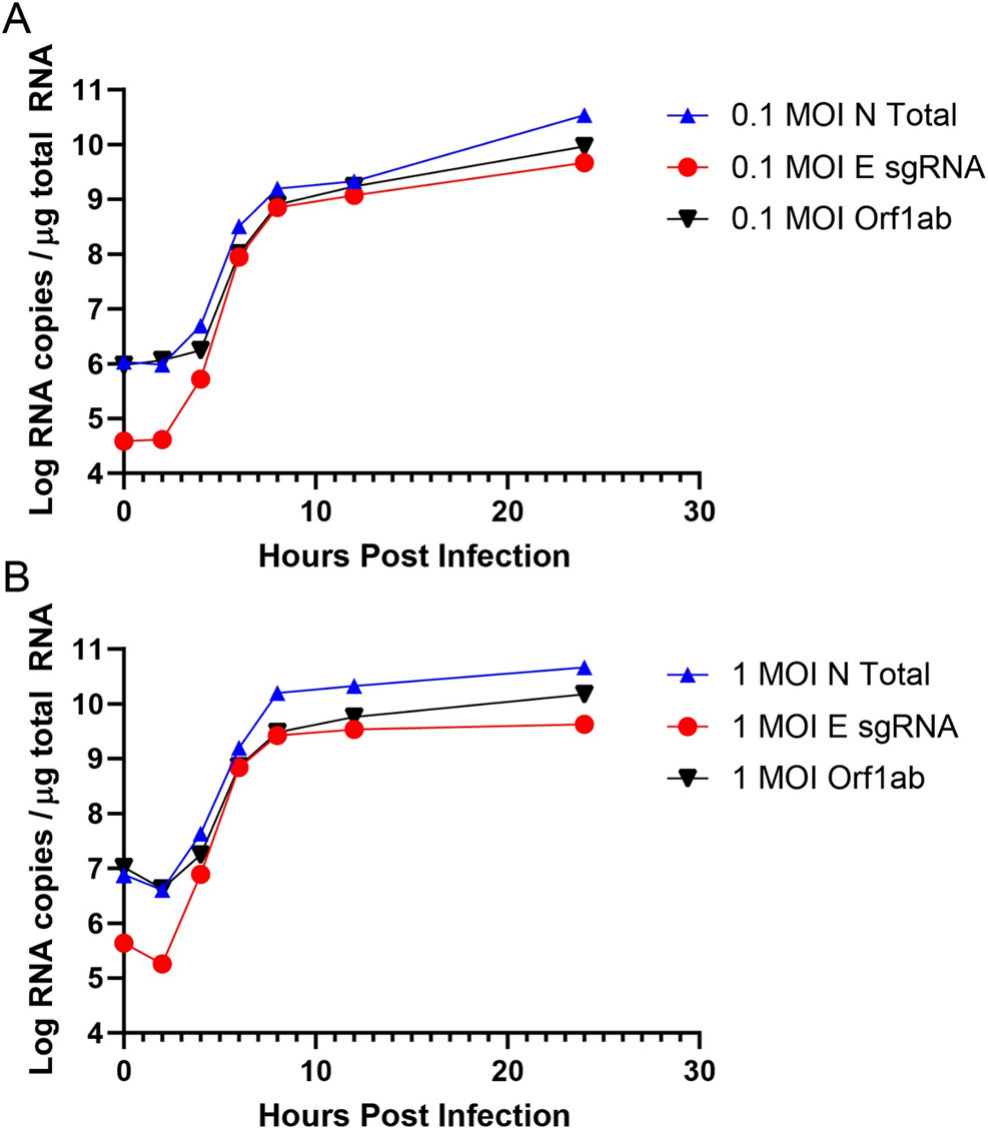
Longitudinal SARS-CoV-2 infection. Vero-E6 cells were infected at 0.1 MOI (A) or 1.0 MOI (B) in 12-well plates. Wells were harvested in triplicate at the following time points: 0, 2, 4, 6, 8, 12, and 24 h postinfection.

We next monitored E sgRNA, N total RNA, and Orf1ab gRNA longitudinally following SARS-CoV-2 infection in Vero-E6 cells. Cells were infected at a multiplicity of infection (MOI) of 0.1 or 1.0 in 12-well plates. At 0, 2, 4, 6, 8, 12, and 24 h postinfection, RNA was extracted for RT-PCR. At 2 h following infection, substantially lower levels of E sgRNA were observed compared with N total RNA or Orf1ab gRNA (Fig. 5), likely reflecting the different molar ratios of sgRNA produced within cells (2, 23). From 2 to 8 h postinfection, all three RNA measurements showed comparable growth as expected (4, 24). Interestingly, after 12 h gRNA appeared to increase at a higher rate than sgRNA, particularly with the 1.0 MOI inoculation, likely reflecting the typically higher levels of gRNA compared with sgRNA in infected cells.

### Monitoring sgRNA and total RNA in NHP SARS-CoV-2 challenge studies.

**FIG 6:**
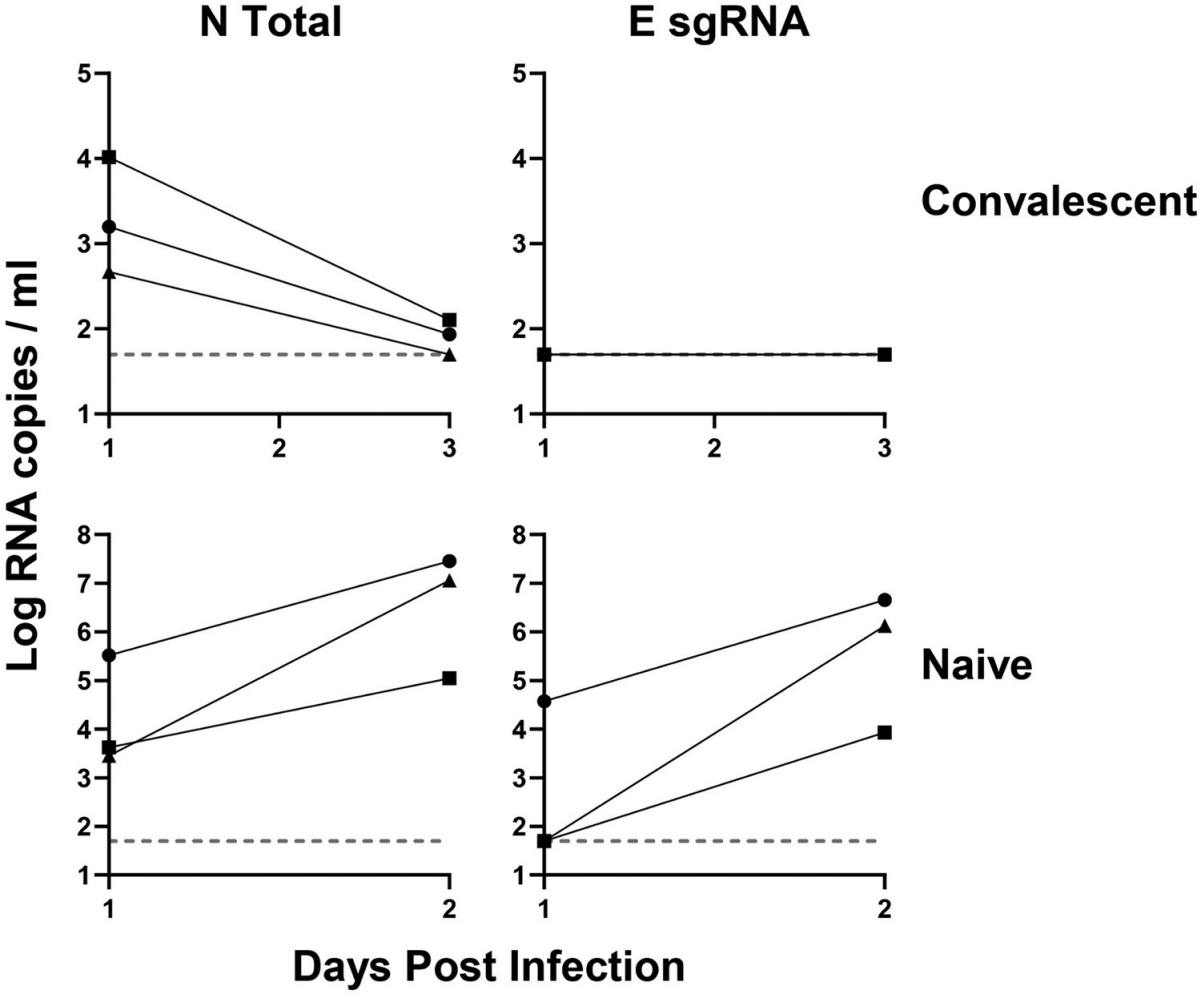
Convalescent NHP SARS-CoV-2 RT-PCR. NHPs were challenged with SARS-CoV-2 and rechallenged 35 days later. RNA extracted from nasal swabs from the rechallenge macaques was run for N total and E sgRNA in naive and the same convalescent animals.

We hypothesized that the E sgRNA assay would be useful for monitoring viral loads in SARS-CoV-2 challenge studies in non-human primates (NHPs), as it should be able to distinguish input challenge virus from newly replicating virus. We have recently reported a study of SARS-CoV-2 infection in rhesus macaques and protection against rechallenge (16). Rhesus macaques were infected with 10^5^ times the 50% tissue culture infective dose (TCID_50_) of SARS-CoV-2 virus intranasally and intratracheally, and were rechallenged with 10^5^ TCID_50_ on day 35 (16). Following rechallenge, there was a median of >10^3^ N total RNA copies/ml in these animals on day 1 that declined by day 3, but undetectable E sgRNA copies/ml (Fig. 6). These data suggest that the N total RNA likely reflected input challenge virus, and that the amount of active virus replication following rechallenge was below the detection limit. In contrast, both N total RNA and E sgRNA were robustly detected in animals by day 2 following primary infection of naive animals (Fig. 6).

**FIG 7:**
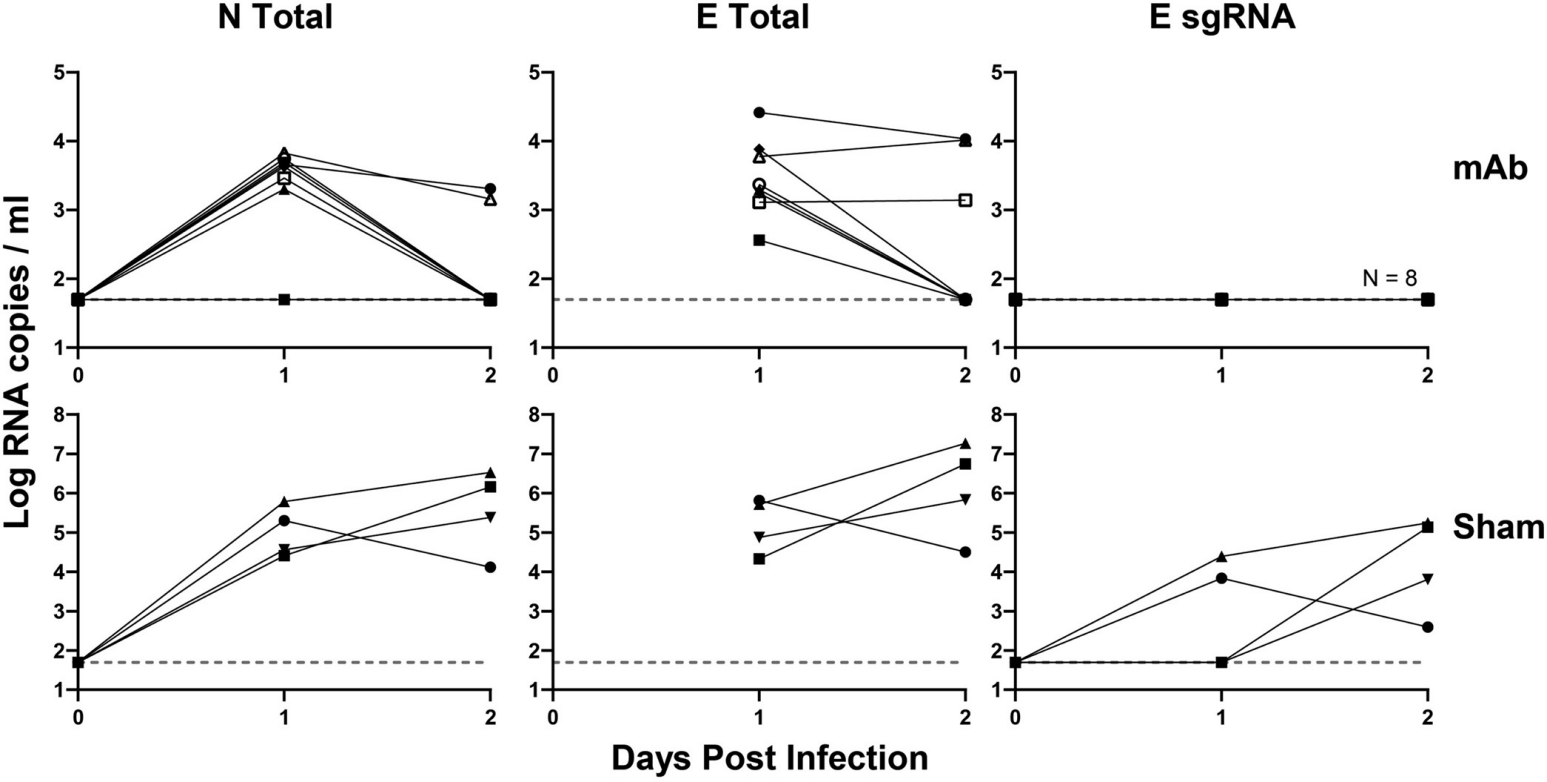
RT-PCR of monoclonal antibody-protected NHPs challenged with SARS-CoV-2. NHPs were given 50 mg/kg of a monoclonal SARS-CoV-2 antibody and then challenged 3 days later with SARS-CoV-2. RNA extracted by BAL fluid was measured for N total, E total, and E sgRNA. Protected macaques (MAb) were compared to unprotected macaques (sham) to demonstrate assay success.

Finally, we evaluated viral loads from macaques that received the monoclonal SARS-CoV-2 antibodies COV2-2196 and COV2-2381. We recently reported that rhesus macaques that received 50 mg/kg intravenously of these SARS-CoV-2 monoclonal antibodies (MAbs) were protected against challenge with 10^5^ TCID_50_ SARS-CoV-2 (25). Low levels of N total and E total RNA were nevertheless detectable on days 1 to 2 following challenge, likely reflecting input challenge virus, whereas E sgRNA was negative at all time points (Fig. 7). The direct comparison of E total RNA and E sgRNA excludes the possibility that the E gene is simply less sensitive than the N gene, given that prior experiments used only N for measuring total RNA.

### Subgenomic RT-PCR viral assay qualification for human use.

Lastly, we qualified the SARS-CoV-2 E sgRNA RT-PCR assay for inter- and intraprecision, assay range, and limit of detection (LOD) using SARS-CoV-2-positive human nasopharyngeal swabs. Tandem assay precision and dilutional linearity were performed to establish the upper limit of quantification (ULOQ) with a percent relative standard deviation (%RSD) of ≤25%, resulting in a ULOQ of 6.57 log RNA copies/ml. LOD determination was based on 2-fold serial dilutions of positive human nasopharyngeal swabs (Table 1). The 95% confidence interval was determined for the lowest detectable RNA copies in the sample dilutions and the LOD defined as the lower limit of this confidence interval, resulting in a LOD value of 2.71 log RNA copies/ml. The assay range was thus determined to have a range of 2.71 to 6.57 log RNA copies/ml. The mean intermediate precision %RSD within this assay range was 4.77% (Table 2). Intra-assay precision within the linear range was established with a predefined ≤25% %RSD and gave an overall precision of 1.85% (Table 3).

**TABLE 1:** Tandem dilutional linearity and intermediate precision for subgenomic viral RNA RT-PCR assay

Subgenomic viral RNA		Log RNA copies/ml^*a*^									
		1	2	3	4	5	6	7	8	9	10
cDNA dilution	Undiluted	4.18	5.12	3.81	–	4.14	3.60	5.23	–	3.68	5.48
	1:1	3.94	4.93	3.65	–	3.85	2.98	4.98	–	3.67	5.19
	1:2	3.57	4.53	3.16	–	3.03	3.54	4.63	–	3.15	4.58
	1:4	3.08	4.23	2.80	–	2.71	–	4.36	–	3.12	4.24
	1:8	–	3.81	2.57	–	–	–	4.01	–	2.75	3.89
	1:16	–	3.42	–	–	2.94	–	3.67	–	–	3.72
	1:32	–	3.00	–	–	–	–	3.21	–	–	3.41
	1:64	–	3.34	2.52	–	2.83	–	2.83	–	2.12	2.41
	1:128	–	–	–	–	–	–	3.27	–	–	2.90
	1:256	–	–	–	–	–	–	2.85	–	–	2.63
	1:512	–	2.26	–	–	–	–	2.93	–	–	–
	1:1,024	–	–	–	–	–	–	–	–	–	–

a The symbol “–“ means undetermined.

**TABLE 2:** Established parameters for the subgenomic viral RNA RT-PCR assay

Parameter^*a*^	Subgenomic RNA
Assay range (log RNA copies/ml)	2.71–6.57
Intermediate precision (%RSD)	4.77%
Intra-assay precision (%RSD)	1.85%
Limit of detection (log RNA copies/ml)	2.71

a RSD, relative standard deviation.

**TABLE 3:** Intra-assay precision for subgenomic viral RNA RT-PCR assay

Operator		Subgenomic viral RNA^*a*^						
		Log RNA copies/ml			GeoMean	Std dev	%RSD	Pass/fail
		Run 1	Run 2	Run 3				
Operator 1								
cDNA Dilution	1:10	5.66	5.50	5.44	5.53	0.11	2.02	Pass
	1:1000	3.63	3.56	3.63	3.61	0.04	1.07	Pass
Operator 2								
cDNA Dilution	1:10	5.52	5.53	5.66	5.57	0.07	1.32	Pass
	1:1000	3.77	3.49	3.80	3.68	0.17	4.70	Pass
Operator 3								
cDNA Dilution	1:10	5.36	5.41	5.33	5.36	0.04	0.75	Pass
	1:1000	3.38	3.44	3.36	3.39	0.04	1.27	Pass

a RSD, relative standard deviation; GeoMean, geometric mean; Std dev, standard deviation.

## DISCUSSION

It is critical for SARS-CoV-2 vaccine and therapeutic studies in rhesus macaques to differentiate input challenge virus from actively replicating virus. Our data demonstrate the potential of measuring sgRNA rather than genomic or total RNA as a more specific measure of replicating virus (4, 16, 18, 24).

SARS-CoV-2 challenge studies administer virus and then sample from the same anatomic sites to assess protective efficacy. RT-PCR assays typically target total RNA, which is present in the input challenge virus. Therefore, an assay that amplifies gRNA (or total RNA) would not be expected to differentiate input or neutralized virus from newly replicating virus. This would make distinguishing vaccine or drug effects difficult at early time points. In contrast, sgRNAs are generated after cell entry in the context of active viral replication. Measuring sgRNA presents a more accurate RT-PCR assay for monitoring the impact of vaccines, MAbs, or other interventions on SARS-CoV-2 virus replication. The E sgRNA assay described here allowed us to differentiate input and replicating virus for assessing the protective efficacy of natural immunity or MAbs in an NHP model (16, 25).

The subgenomic E (sgE) gene was used to measure sgRNA levels in this work (17). In the future, it may be reasonable to explore other sgRNAs in similar assays to increase sensitivity. In particular, the sgE gene is transcribed at a lower level than the subgenomic N gene (2, 21). In summary, total RNA or gRNA may not be an optimal measure of protective efficacy following SARS-CoV-2 challenge, as it includes input challenge virus; therefore, sgRNA may be more relevant for measuring actively replicating virus *in vivo*. These findings are important for the evaluation of SARS-CoV-2 prophylactic and therapeutic agents.

## MATERIALS AND METHODS

### Synthetic genes.

Genomic and subgenomic genes were synthesized based on the SARS-CoV-2 USA-WA1/2020 genome (GenBank MN985325.1) and following the schematic previously described (17). All subgenomic genes contain the SARS-CoV-2 leader sequence followed by the transcriptional regulatory sequence (TRS) and the structural genes spike (S), envelope (E), membrane (M), and nucleocapsid (N). Genes were synthesized by Integrated DNA Technologies and confirmed by sequencing. For RNA targeting assays, standard curves were generated for each synthetic gene by cloning into a pcDNA3.1 expression plasmid then *in vitro* transcribing using an AmpliCap-Max T7 High Yield Message Maker kit (Cellscript). Log dilutions of the resulting *in vitro*-transcribed RNA were prepared.

### RT-PCR.

The RNA transcripts were reverse transcribed using Superscript III VILO (Invitrogen) according to the manufacturer’s instructions. A TaqMan custom gene expression assay (Thermo Fisher Scientific) was designed to specifically target each genomic and subgenomic gene. The samples were run in duplicate in a QuantStudio 6 Flex real-time PCR system (Life Technologies) using the following conditions: 95°C for 20 s then 45 cycles of 95°C for 1 s and 60°C for 20 s. For all RT-PCR runs the following quality control (QC) acceptance range for standard curves must be met: R^2^ > 0.98, efficiency 90 to 110%, and slope −3.1 < x > −3.6. The amplified RT-PCR products were run on 0.8% agarose gels for confirmation of subgenomic E amplification.

**Primer sequences (Table 4).** RT-PCR was performed on the E subgenomic gene using the leader forward primer sgLeadSARSCoV2-F (CGATCTCTTGTAGATCTGTTCTC) and the complementing probes and reverse primers as follows: E_Sarbeco_R, ATATTGCAGCAGTACGCACACA, and E_Sarbeco_P1 (probe): VIC-ACACTAGCCATCCTTACTGCGCTTCG-MGBNFQ (17). RT-PCR was also performed on the ORF1ab gene using the following: SARS-CoV2.ORF1ab.F, GGCCAATTCTGCTGTCAAATTA; SARS-CoV2.ORF1ab.R, CAGTGCAAGCAGTTTGTGTAG; and SARS-CoV2.ORF1ab.P, FAM-ACAGATGTCTTGTGCTGCCGGTA-BHQ1. The complementing N total viral RNA gene primers and probe were used as describe previously (15).

**TABLE 4:** Primers and probes for RT-PCR

Gene	Oligonucleotide	Primer/probe	Sequence 5′ to 3′
Subgenomic Envelope (E)	sgLeadSARSCoV2-F	Forward Primer	CGATCTCTTGTAGATCTGTTCTC
	E_Sarbeco_R	Reverse Primer	ATATTGCAGCAGTACGCACACA
	E_Sarbeco_P1	Probe	VIC-ACACTAGCCATCCTTACTGCGCTTCG-MGBNFQ
Envelope (E)	E_Sarbeco_F	Forward Primer	ACAGGTACGTTAATAGTTAATAGCGT
	E_Sarbeco_R	Reverse Primer	ATATTGCAGCAGTACGCACACA
	E_Sarbeco_P1	Probe	FAM-ACACTAGCCATCCTTACTGCGCTTCG-BHQ1
Nucleocapsid (N)	2019-nCoV_N1-F	Forward Primer	GACCCCAAAATCAGCGAAAT
	2019-nCoV_N1-R	Reverse Primer	TCTGGTTACTGCCAGTTGAATCTG
	2019-nCoV_N1-P	Probe	FAM-ACCCCGCATTACGTTTGGTGGACC-BHQ1
ORF1ab	SARS-CoV2.ORF1ab.F	Forward Primer	GGCCAATTCTGCTGTCAAATTA
	SARS-CoV2.ORF1ab.R	Reverse Primer	CAGTGCAAGCAGTTTGTGTAG
	SARS-CoV2.ORF1ab.P	Probe	FAM-ACAGATGTCTTGTGCTGCCGGTA-BHQ1

### RNase A-treated SARS-CoV-2 *in vitro* infection.

SARS-CoV-2 virus stocks were diluted to MOIs of 0.1 and 1.0 in infection medium and treated with 200 μl or 20 μl of RNase A (Sigma: R4642) for 1 h at 37°C. The infection medium negative control was also treated with 200 μl or 20 μl of RNase A for 1 h at 37°C. SARS-CoV-2-treated stocks were then lysed with 500 μl of TRIzol reagent. Total RNA was extracted from cells using a QIAcube HT (Qiagen) and RNeasy 96 QIAcube HT kit (Qiagen). RNA was reverse transcribed into cDNA using superscript VILO (Invitrogen). RT-PCR was performed as described above.

### *In vitro* SARS-CoV-2 infection.

Vero-E6 cells were seeded in 12-well plates (Corning) at 300,000 cells per well the day prior to infection in growth medium (DMEM, 5% fetal clone II, 1% antibiotic-antimycotic). On the day of infection, SARS-CoV-2 infectious viral particles were treated with 25 units of RNase H (Promega M4281) for 1 h at 37°C. Cells were then infected in triplicate wells at a 0.1 or 1.0 multiplicity of infection (MOI) of RNase H-treated SARS-CoV-2 and RNase H-treated infection medium (DMEM, 2% fetal clone II, 1% antibiotic-antimycotic) negative control for 1 h at 37°C. Following infection, Vero-E6 cells were thoroughly washed three times with 1 ml of sterile 1× phosphate-buffered saline (PBS) and 500 μl of infection medium was replaced in each well. Cells were then harvested at 0, 2, 4, 6, 8, 12, and 24 h postinfection. Prior to harvesting each time point, cells were twice washed with 1 ml of sterile 1× PBS, lysed with 300 μl of TRIzol reagent, and were immediately frozen. Total RNA was extracted from cells using a QIAcube HT (Qiagen) and RNeasy 96 QIAcube HT kit (Qiagen). RNA was reverse transcribed into cDNA using superscript VILO (Invitrogen). RT-PCR was performed as described above.

### NHP monoclonal antibody studies.

As part of the study, 12 healthy female and male rhesus macaques (Macaca mulatta) of Indian origin ranging in weight from 5 to 15 kg were studied as previously described (25). The monkeys were randomly allocated into three groups as follows: group 1, anti-SARS-CoV-2 MAb COV2-2196 (*n* = 4); group 2, anti-SARS-CoV-2 MAb COV2-2381 (*n* = 4); group 3, sham IgG (*n* = 4). The animals were given one dose of 50 mg/kg of anti-SARS-CoV-2 antibody or sham isotype intravenously on day −3. All animals were subsequently challenged with 10^5^ TCID_50_ SARS-CoV-2, administered as 1 ml by the intranasal route and 1 ml by the intratracheal route on day 3 post antibody infusion. All animal studies were conducted in compliance with all relevant local, state, and federal regulations and were approved by the Bioqual Institutional Animal Care and Use Committee (IACUC).

Viral RNA was quantified using an RT-PCR assay targeting the SARS-CoV-2 nucleocapsid and subgenomic envelope genes. RNA was isolated from nasal swabs and BAL fluid collected from macaques using the cador Pathogen 96 QIAcube HT kit and a Qiacube HT (Qiagen). RT-PCR was performed as described above.

### NHP rechallenge model.

Three outbred Indian-origin adult male and female rhesus macaques (Macaca mulatta), 6 to 12 years old, were used to set up the RT-PCR assays, which were previously reported (16). All animals were housed at Bioqual, Inc. (Rockville, MD). All animals were inoculated with SARS-CoV-2 at a total dose of 10^5^ TCID_50_ on day 0. The dose was administered as 1 ml by the intranasal (i.n.) route (0.5 ml in each nare) and 1 ml by the intratracheal (i.t.) route. On day 35 following challenge, animals were rechallenged with SARS-CoV-2 with the same dose utilized in the initial challenge. All animal studies were conducted in compliance with all relevant local, state, and federal regulations and were approved by the Bioqual Institutional Animal Care and Use Committee (IACUC). RT-PCR was performed as described above.

### Subgenomic assay qualification.

Reverse transcribed cDNA (derived from pooled RNA extracted from the nasopharyngeal swab samples of SARS-CoV-2-infected individuals with >10^7^ viral copies/ml) was tested undiluted and serially diluted (in log dilutions) to assess linearity and intermediate precision for the subgenomic viral RNA assay. Three different operators performed these assays over three different days for each assay run. The highest value of the sample dilution range with a precision of relative standard deviation (RSD) ≤25% was used to define the upper limit of quantification (ULOQ). To determine intra-assay precision, two cDNA dilutions within the linear range were selected to approximate high and low levels of the ranges. At these approximate high and low levels, predefined intra-assay precision of RSD ≤25% was met by each individual operator.

**Limit of detection.** Serial dilutions of 10 individual SARS-CoV-2-positive cDNA samples from nasopharyngeal swabs derived from positive individuals were tested in 2-fold dilutions. Within each dilution series, the last positive value or last positive value prior to sample becoming undetectable was used in LOD calculations. Any positive values observed beyond the first undetectable result in a dilution series were considered not valid. The 95% confidence interval was obtained for these samples and the LOD defined as the lower limit of this confidence interval, reported as log RNA copies/ml.
